# Improving Student Engagement in the Study of Professional Ethics: Concepts and an Example in Cyber Security

**DOI:** 10.1007/s11948-017-9904-4

**Published:** 2017-04-11

**Authors:** John D. Bustard

**Affiliations:** School of Electronics, Electrical Engineering and Computer Science, Queen’s University, Belfast, BT9 5AH UK

**Keywords:** Ethics, Cyber security, Case studies, Psychology, Curriculum design

## Abstract

In spite of the acknowledged importance of professional ethics, technical students often show little enthusiasm for studying the subject. This paper considers how such engagement might be improved. Four guiding principles for promoting engagement are identified: (1) *aligning teaching content with student interests*; (2) *taking a pragmatic rather than a philosophical approach to issue resolution*; (3) *addressing the full complexity of real*-*world case studies*; and (4) *covering content in a way that students find entertaining*. The use of these principles is then discussed with respect to the specific experience of developing and presenting a master’s module in Ethical and Legal Issues in Cyber Security at Queens University Belfast. One significant aspect of the resulting design is that it encourages students to see ethical issues in systemic terms rather than from an individual perspective, with issues emerging from a conflict between different groups with different vested interests. Case studies are used to examine how personal and business priorities create conflicts that can lead to negative press, fines and punitive legal action. The module explores the reasons why organisations may be unaware of the risks associated with their actions and how an inappropriate response to an ethical issue can significantly aggravate a situation. The module has been delivered in three successive years since 2014 and been well received on each occasion. The paper describes the design of the module and the experience of delivering it, concluding with a discussion of the effectiveness of the approach.

## Introduction

Most courses in engineering and other technical subjects include some coverage of professional ethics. This can range from a block of lectures for undergraduates to a full module at postgraduate level. Usually this is mandatory, as all students are expected to have a good appreciation of the ethical issues that can arise in professional life, and an understanding of how such issues might be addressed. The importance of teaching ethics to university students is particularly emphasised by professional bodies in the context of accreditation (Ocone 2013).

In contrast to this apparent importance, however, many students approach the study of professional ethics with little enthusiasm. For example, Coldwell (2000) notes, in describing her experience of teaching an ethics module by distance learning, that “The technically-oriented students perceived [the module] as a hurdle they had to clear in order to qualify for their degree rather than one where they had an opportunity to learn some new information technology related skill.” This is unfortunate, as a lack of interest implies less than full engagement, meaning that the subject will be less well studied, and the students less competent when they graduate.

The purpose of this paper is to examine how students might be encouraged to engage more fully in the study of professional ethics. The work was triggered by the need, in 2014, to design a module in Ethical and Legal Issues in Cyber Security for a new Masters course at Queens University Belfast. The module received particular management attention within the course because an employer survey on potential course content, carried out during its design phase, highlighted ethics skills above any other cyber security competence.

Student opinion of the ethics module would be judged through a standard university questionnaire, submitted anonymously and collated by an external organisation. Here students score a range of performance statements on a scale of 1 (strongly disagree) to 5 (strongly agree). Open ended questions are also included to identify specific examples of good teaching practice, areas for improvement and any general comments. The performance statements include “The lecturer explained new terms, concepts and principles clearly,” and “The lecturer made the subject interesting.” Giving equal weight to each statement, and averaging the scores received, the University has set an aspirational target of 4.2 for each module. The goal, therefore, was to design and deliver an ethics module that received a score at this level or higher.

It was recognised at the outset that ensuring student engagement would be a significant step towards developing a successful module. Ways to promote engagement were considered, with relevant factors identified from a number of sources. These included the general education literature, as well as books and articles dealing directly with professional ethics. The factors that emerged were then distilled into a set of four guiding principles to be used in the design of the module. These were:

1. Aligning teaching content with student interests.
2. Taking a pragmatic rather than a philosophical approach to issue resolution.
3. Addressing the full complexity of real-world case studies.
4. Covering content in a way that students find entertaining.

The next section of the paper explains these principles, illustrating the discussion with respect to the content of the cyber security ethics module. Details of the delivery of the module are then discussed, followed by some final thoughts on the effectiveness of the approach.

## Engagement Principles

In education, ‘engagement’ is essentially the measure of a student’s participation in a learning task, with those engaged showing “sustained behavioural involvement in learning activities accompanied by a positive emotional tone” (Skinner and Belmont 1993). The purpose of this section is to consider a set of four general principles that might help encourage engagement in teaching professional ethics to students studying a cyber security master’s course.

### Selecting Content Relevant to Student Interests

For students to engage fully in the study of any subject they need to find it interesting (Schiefele 1991). With professional ethics, some may have an intrinsic interest but most are likely to need encouragement. In particular, one initial barrier, as highlighted by Harris et al. (2013), is that many feel that they already have a good informal understanding of the subject, asking, in effect, “Why should I study ethics? I am an ethical person.’’

The first lecture of the ethics module therefore starts with an explanation of why the module exists and why it is compulsory. The most important factor is the catastrophic impact that mishandling ethical issues can have on organisations. This is emphasised by professional bodies, such as the Royal Academy of Engineering (2017) and perhaps more significantly, by local companies, where many of cyber security students will be seeking employment. The latter message was reinforced with results from the survey used to help define requirements for the course, as mentioned in the introduction.

The general relevance requirement for an ethics module in cyber security is that the students should feel that the content is closely aligned with the subject field. Coverage therefore includes a consideration of best practice when dealing with common cyber security tasks such as *responsible disclosure*, *handling forensic evidence*, *employee monitoring* and *security research*. Relevance is emphasised as each new topic is introduced, highlighting the practical losses due to mishandling a situation.

The focus on relevance also extends to the assessed material for the course, across coursework and an examination. All coursework exercises and the majority of examination questions require the students to respond to cyber security based scenarios of the type that might be encountered in the workplace.

Cyber security is a field that is heavily influenced by ‘scandal’—*an action or event regarded as morally or legally wrong and causing general public outrage* (Oxford Living Dictionaries 2017). The module considers how scandals can emerge. One source is a failure to protect organisations and individuals adequately. An example is the TalkTalk Data Breach (ICO 2016a), where cyber security vulnerabilities were exploited by hackers to steal the personal details of over 156,000 customers, including over 15,000 bank account and sort code records. Cyber security scandals receive particular attention when they occur at a national level. For example, the NSA leaks by Edward Snowdon (Wright and Kreissl 2013) revealed secret global surveillance programmes run by the US National Security Agency in collaboration with Australia, Canada, New Zealand, and the United Kingdom.

Scandals can also emerge from the inappropriate use of security software. For example, in the Webcam-gate case (District Court 2010), a school provided laptops to children with hidden surveillance software installed on it, used to secretly capture images of children in their homes. Another example is the Sony Rootkit case (Stanley 2008), where Sony included copy protection software with some music CDs which modified the Windows operating system. The changes were initially impossible to uninstall and introduced security vulnerabilities. Sony ultimately lost a class action lawsuit and was forced to issue a full product recall.

As a professional, a cyber security expert has to focus as much on minimising the risks of such scandals as they do in mastering the technicalities of encryption or network analysis. A key part of such risk analysis is understanding that some actions that can be taken to secure systems can create as great a risk to an organisation as the cyberattacks they are trying to prevent.

In practice, it has not been difficult to find relevant content for the ethics module as cyber security scandals are commonplace, with new examples appearing frequently. This availability makes it practical to refresh the module with a few new studies each year, increasing the immediacy of the material and so potentially capturing student interest to a greater extent.

### Taking a Pragmatic Rather Than a Philosophical Approach to Issue Resolution

Koehler (2003) notes that “Ethics are generally perceived to derive from and serve as the application of moral principles.” Such statements imply that there is a set of agreed principles and values, which if followed will result in praise or at least a lack of criticism from society. From a pragmatic perspective, however, it is arguably more important to appreciate how groups within society are likely to react to potentially scandalous situations than to have an academic understanding of what is moral.

Most cultures do not have a single moral authority but an ecosystem of competing ideologies that can use moral outrage as a tool to increase power and influence (Alinsky 2010). As a result, a simplistic moral code is unlikely to provide sufficient perspective to protect organisations from scandal. This is particularly the case when such organisations deal with complex competing priorities such as security and privacy. To address this concern, instead of attempting to increase the students’ skills in philosophical thinking or rhetorical debate, they are given many practical examples of how acting in a way that others consider unethical has led to significant losses. The intention is for students to learn from examples rather than adopt a particular moral framework.

The course was designed to avoid the competitive, argumentative format that philosophical or moral debate frequently uses. Psychological studies suggest that such discussion typically serves to intensify the beliefs of each side rather than help achieve a pragmatic resolution to an issue (Nyhan and Reifler 2010). The module therefore avoids the concept of a ‘true’, or ‘best’, or even ‘balanced’ response to a given situation. Instead it encourages a risk oriented approach, with an examination of the negative reactions that can occur in each situation used to determine an appropriate course of action.

Similarly, when it came to teaching cyber security laws, the focus was not on the literal wording of the legislation but on an explanation of why laws were introduced and how they are used in practice. In particular, through case studies, the course shows that the enforcement of law can be affected by public opinion and political influence (Home Affairs Committee 2012).

The module is intended to describe how the world is; not how it should be. Students are therefore encouraged to explore any viable response to an ethical dilemma, even if it might be considered amoral by some. In coursework, the students are assessed on the reasoning they provide, including the thoroughness and practicality of the evidence offered, rather than any judgement of moral value.

During delivery, the practical difficulties that students can face in handling ethical issues in practice are acknowledged. This is particularly the case when discussing sensitive situations such as whistleblowing or inappropriate employee monitoring (Bustard 2013). In such circumstances, there may be significant organisational pressure not to intervene, with individuals having insufficient power to raise concerns without some risk of negative personal consequences. The goal of the module is not to moralise and, in effect, coerce students into acting against their interests, but to allow them to decide for themselves how best to act in any given situation, following a suitably thorough analysis of possible courses of action.

In terms of underlying theories, the module includes a consideration of psychological biases. The purpose here is to draw attention to the types of situation in which people can lose sight of how others might perceive their actions. In particular, the module highlights our human tendency to do harm to each other, to permit such abuse, and to rationalise such actions. These traits are illustrated through seminal psychology research, including the Stanford Prison Experiment (Zimbardo et al. 2000), Miligram’s Obedience Experiment (2009) and the Robbers Cave Study (Sherif 2010).

### Addressing the Full Complexity of Real-World Case Studies

Professional ethics is typically taught through the analysis of case studies (Harris et al. 2013). There is, however, some tension between the focus by professional bodies on studies involving decision making by individuals and the need to address the wider complexity that is often present in real-world situations. Bucciarelli (2008) argues that “In focusing solely on an individual agent’s possible courses of action [many current case studies] oversimplify; they are not a valid abstraction.” He suggests instead that what is needed are studies that reflect “a more expansive and critical study of engineering, including its social/political dimensions.” In effect, this argues for a systemic approach (Senge 2006) to understand the social and political dynamics of ethical issues.

Systemic thinking is particularly relevant to cyber security where activity in this area often raises many social and political questions. For example, cyber security techniques can easily be abused (District Court 2010) and there are general concerns among the public about how such technology can lead to an invasion of privacy (TNS Opinion & Social 2015; Bustard 2015). With sophisticated developments in artificial intelligence and increasingly pervasive technology, such as the Internet of Things, the temptation to monitor and manipulate individuals is greater than ever (Baldini et al. 2016). While there have been efforts to establish good practice for such technology (DC-IoT 2016) the conflict between the economic benefits of gathering data and the consumers’ desire for privacy will almost certainly result in scandals, with corresponding losses to organisations and their members. This is particularly the case as governments and legal entities introduce more punitive measures to address such scandals. For example, the Federal Trade Commission (FTC) in the US (Farrel et al. 2012) is imposing large fines on organisations violating data protection legislation. New European Union legislation is likely to result in similar punitive fines as the legislation increases the liability of those who collect personal data (Hon 2016). Such restrictions are unlikely to be lifted while public concern about privacy remains high (TNS Opinion & Social 2015).

Likewise, as cyber security attacks become increasingly political, the risk of overreach and the resulting possibility of scandal are likely to continue. State sponsored attacks, such as the hacking of Hillary Clinton’s email server (FBI 2016), may well increase, with other nations then prompted to invest heavily in protective measures. For example, the UK government has recently allocated £1.9 billion to improve cyber defences, including support for retaliation in the face of attack (BBC News 2016).

Overt cyber offences committed by criminals are also scandalous. However, the module does not dwell on these issues. Instead it focuses on the grey areas of cyber security legislation and practice. These are the areas where organisations may not realise that their actions might cause a scandal.

### Covering the Content in a Way that the Students Find Entertaining

There is an obvious link between student engagement and the extent to which they enjoy the experience (Malone and Lepper 1987). With the teaching of professional ethics so much based on a consideration of case studies, there is an opportunity for the lecturer to promote engagement by taking a storytelling approach to each study (Alterio and McDrury 2003). The nature of the often dramatic incidents in cyber security make such presentations inherently entertaining. Engagement can be further strengthened by focusing on the personalities and motivations of the individuals involved in each ethical issue. Such stories are memorable, helping the students retain knowledge of the key concepts in each case. Storytelling can also be enjoyable for the lecturer, leading to enthusiasm for the material presented, which in itself promotes student engagement.

The next section describes the design and delivery of the module developed around these principles.

## Module Design and Delivery

The Ethical and Legal Issues in Cyber Security master’s module began in 2014 and has been presented three times so far, with 10, 18 and 30 students attending in successive years. The module was originally covered over a 12-week semester, with one 2-h lecture each week and three 3-h seminars run in support of coursework guidance and revision of the taught material. For 2016–17, the module was restructured into a single teaching block, delivered over five days. This change was made to facilitate students who had full time work commitments. The sub-sections below describe the most recent delivery.

In relation to legal issues, the module focuses on United Kingdom (UK) legislation, as this is the most relevant jurisdiction for the majority of students involved. However, the module also includes many case study examples from the United States (US), particularly where legal or governmental actions have shaped UK policy. Many scandals are also international in scope, in which case the losses and legal rulings in the main jurisdictions are described. Relevant European legislation is also discussed, such as the new General Data Protection Regulation (ICO GDPR 2016b).

### Day 1

The first day of the module encourages the students to think beyond a simple ‘good guys’ and ‘bad guys’ view of cyber security. The Sony Rootkit case study is used to show how individuals who may believe they are doing the right thing can create a scandal (Stanley 2008). The approach used by Sony is an attempt to implement a technical solution to the illegal copying of their music—a practice that was not being effectively controlled at that time. It is plausible that those developing the technology felt they were justified in deploying such a solution and were solving an important problem that others were ignoring. The resulting scandal was, however, detrimental to Sony, both in terms of the effect on their image and the cost of legal action, followed a full product recall.

The dynamics of scandals are also discussed. It is shown how different groups within society, such as politicians, journalists and activists, shape and intensify scandals for their own purposes. The Prestel hacking case is then used to examine these issues and to illustrate how scandals can lead to the introduction of new laws (Leyden 2015). The Prestel system was a precursor to the World Wide Web, a dial up information service that included electronic mail and basic ecommerce facilities. The Prestel case occurred when its owners and the Metropolitan Police prosecuted two journalists who had identified vulnerabilities in the system and were publically critical of its security. The case ultimately resulted in the first piece of UK cybercrime legislation, the Computer Misuse Act.

The first day concludes with an examination of discrimination law and psychological theories about why discrimination is so common. In particular, this includes a consideration of why it may be difficult for people to realise they are indirectly discriminating through their actions.

### Day 2

The second day starts by examining the Computer Misuse act in detail. This includes an analysis of areas of ambiguity as to when the law is broken, especially where it is unclear whether authorisation has been granted.

The second half of the day considers issues arising from ‘social engineering’ techniques, which in the context of information security means *“*the use of deception to manipulate individuals into divulging confidential or personal information that may be used for fraudulent purposes*”* (Oxford Living Dictionaries 2017). Such techniques are a key component in many cybercrimes but are not explicitly covered by computer misuse legislation. The discussion includes an overview of the Fraud Act, which is the UK law that is broken during social engineering attacks.

The Hewlett-Packard Spying scandal is then discussed to illustrate how private investigators may be motivated to engage in social engineering (Kaplan 2006). The HP case began when the chairwoman of HP ordered an investigation into the source of discussion leaks at a corporate retreat attended by board members and the new CEO. The resulting investigation made use of a number of social engineering techniques to gain access to private communications between journalists and board members. The HP case is an example of a situation under which such actions may be prosecuted. It is particularly significant, as it led to the introduction of new legislation explicitly making such activity illegal in the US.

The discussion of social engineering continues with an investigation by the UK Home Affairs Committee that criticised the organisational culture within the private investigation profession (Home Affairs Committee 2012). As with the Prestel case, this is a situation where individuals were prosecuted selectively for behaviour that is common practice in order to change the public perception of what is legitimate.

The day concludes with an examination of international legislation and professional codes of conduct relating to cybercrime. This includes an examination of how governments, police forces and organisations may pass on responsibility for cyber security to others rather than addressing issues themselves. This section also highlights how professionals may find themselves in the difficult position of balancing privacy and security concerns, and the risks associated with making the wrong decision.

### Day 3

The third day focuses on data protection and privacy issues. The Webcam-gate scandal is used as an example of how groups of individuals can fail to realise that they are engaged in a highly inappropriate invasion of privacy. In the Webcam-gate case, many school employees were aware of the secret surveillance of children in their homes but failed to take action to stop it.

The day continues with an examination of human rights legislation and UK cases where challenges brought to the European court of human rights have been upheld. The current state of legal challenges to the UK Government Communications Headquarters (GCHQ) surveillance practices are also discussed. This leads on to an overview of the new Investigatory Powers Act within the UK which provides a legal basis for the surveillance practices of GCHQ and other government agencies.

The afternoon focuses on the UK Data Protection Act and the new European General Data Protection Regulation. The Safari Cookie case is used to show how data protection issues that are seemingly minor can result in significant fines under certain circumstances (Farrell et al. 2012). This scandal arose when Google incorrectly stated that their user profiling technology was only active for Safari browser users when logged into a Google account. This resulted in one of the largest data protection fines issued by the Federal Communications Commission (FCC). Examples and statistics from the UK Information Commissioner’s Office are then used to show likely causes and fines for different forms of data protection issue.

The day concludes with a discussion of approaches to reducing privacy issues such as Privacy by Design (Gürses et al. 2011) and the use of a privacy impact assessment (Wright and De Hert 2012).

### Day 4

The fourth day focuses on cyber security practices, including employee monitoring and surveillance. Cyber security professional practices are discussed, including *responsible disclosure*, *penetration testing*, *digital forensics* and *security research* techniques, such as honeypots.

The Brett McDanel case is used to highlight the risks of revealing security vulnerabilities without following responsible disclosure practices (Freeman 2007). Brett McDanel was wrongly jailed for 16 months for revealing a security vulnerability in his former employers email service. He was later exonerated on appeal after it was clarified that revealing such vulnerabilities did not in itself constitute a damaging attack on a computer system.

In the afternoon, insider threats and employee monitoring are examined, with relevant legislation and advice from the UK Information Commissioner’s Office (ICO) discussed. This area has limited evidence to support it because most employment issues are settled in tribunals. Such cases often involve settlements with a condition of confidentiality. This makes it very difficult to properly assess risks for organisations and individuals, and greatly reduces the reputational deterrent that would normally follow from breaking the law. To ensure that this section of the course reflected plausible risks and likely outcomes, a lawyer with experience litigating in privacy and technology cases provided a guest lecture and commentary on the issues raised throughout the day.

### Day 5

The final day of the course covers issues of ethical research, whistleblowing, scandal management and hacktivism, where hacktivists are those who “gain unauthorized access to computer files or networks in order to further social or political ends” (Oxford Living Dictionaries 2017).

A variety of ethics issues relating to research are covered include those arising from human experimentation. Relevant cyber security issues are discussed including the controversies surrounding the use of social media data without explicit consent.

The psychology and dynamics of whistleblowing are then examined. This includes a case study where the US Food and Drugs Administration (FDA) used cyber security software to monitor whistleblowing employees (Nakashima and Rein 2012), including their communications with journalists and members of congress. The practical statistics of whistleblowing in the UK are also examined, using a survey produced by the charity Public Concern at Work (PCAW 2014).

The Public Interest Disclosure Act is then discussed, which allows for compensation if organisations retaliate against whistleblowing employees. The practical difficulties of proving retaliation are also mentioned. This leads to a wider discussion about what might make whistleblowing effective and includes suggestions for how to minimise the risks of retaliation. Examples from earlier case studies are used to support the discussion, as well as the cases of Sherron Watkins at Enron and Cynthia Cooper at Worldcom (Lacayo and Ripley 2002).

The day continues with an analysis of how organisations have responded to scandals. A range of formal apologies published after data breaches are examined. These include the two very different responses from Sony before and after losing their class action lawsuit over Rootkit, and the changes made to their music CD terms and conditions as a result of this case (Stanley 2008).

The final topic considered is hacktivism. It starts with an overview of the early conflicts between the Church of Scientology and the Cult of the Dead Cow hacker group (Ratte 1995). This occurred when an ex-Scientologist who was critical of the church posted internal information about the organisation to newsgroups. The church attempted to intimidate this individual and supporters of the church attempted to interfere with the newsgroup to prevent dissemination of the information. This led to a hacker group declaring the church a legitimate target for attacks and started a long-running conflict between hacktivists and the church.

The case of the Project Chanology conflict that gave rise to Anonymous as a movement (Leyden 2008) is then discussed. This occurred when an internal Scientology video was published on Youtube and other video sharing sites. The video showed the actor Tom Cruise talking about the powers and the importance of Scientology members. The church took legal action to attempt to stop the spread of the video and this in turn led to a new hacktivist group opposing them. Through criticism of the church in many media outlets, and ultimately through public protests, Anonymous became a popular and influential hacktivist organisation.

The HBGary hack is then examined. This occurred when Aaron Barr, the CEO of a subsidiary of HBGary, claimed that he could identify members of Anonymous. This resulted in a cyberattack against HBGary, revealing many internal documents, including proposals to discredit Wikileaks using false information and plans to intimidate Glenn Greenwald, a journalist specialising in civil rights issues. The documents also included a proposal for large scale illegal hacking to create a botnet of compromised computers (Krebs 2011). Each case study is analysed to show how the attacked side escalated or mitigated the attacks.

The module concludes by describing the case of Aaron Swartz, a cofounder of Reddit and activist who committed suicide while being investigated by the FBI (Knappenberger 2014). He faced thirteen charges resulting from an attempt to gather a large quantity of academic research papers, with the suggested intention of releasing them to the public for free. The FBI had decided to make an example of him, and the drawn out prosecution largely bankrupted him and his parents.

### Coursework

Assessment of the module is through a 3-h examination, and three pieces of coursework, with a relative weighting of 10%, 60% and 30%. The first exercise is set before the students face the taught material, as a way of encouraging them to think about the complexity of real-world ethical situations. Each student is given a different cyber security related scandal, asked to analyse it, and then create a 10 min video describing the timeline of events, involvement of key stakeholders, and possible motivations behind their actions. The purpose of the exercise is to encourage students to think beyond a simple ‘good guys’ and ‘bad guys’ narrative of events. In particular, it is intended to help them appreciate how different groups within society interact and shape scandals.

In the second piece of coursework, each student is asked to produce a privacy impact assessment (PIA) in which he/she analyses the privacy implications of an assigned product or service, following guidelines covered in lectures. The students are divided into teams of four or five. Each person in the team is given a different scenario, with the other team members representing particular stakeholders and completing a questionnaire based on each of those roles. The questionnaire responses are intended to help identify a set of risks and ways of addressing those risks, contributing to the completion of the PIA.

The third and final piece of coursework builds directly on the second exercise. Each student is given a specific ethical issue related to their assigned product or service. In the role of a whistleblower, they then have to construct an email reporting the issue, and then in a management role, create a matching press release. Equal weight is given to each part. Both the whistleblowing email and press release take a similar form. They are intended to be realistic, professional communications of the type created in high stress situations, typical of what the students may face at some point in their career. The guidance given encourages students to imagine how others might judge and react to their actions. Students are explicitly told that they are assessed on the depth and justification of their reasoning rather than the way in which they chose to address the scandal.

## Reflection and Conclusions

The purpose of this paper has been to consider general ways of improving student engagement in the study of professional ethics. Four high-level guiding principles were identified. These were discussed with respect to the design and delivery of a specific master’s module in Ethical and Legal Issues in Cyber Security at Queens University Belfast.

The key characteristics of the resulting module are that:

• It stresses the importance of professional ethics by illustrating the substantial impact that the mishandling of ethical issues can have on an organisation.
• It promotes student interest through the use of an entertaining storytelling approach to the consideration of ethical issues, based around high-profile cyber security scandals.
• It emphasises the practical need to address an ethical issue as a systemic problem, acknowledging that although the issue may be triggered by an individual’s moral concerns, resolution will require a system-wide investigation of the stakeholders, their likely motivations and their practical power to influence the outcome.

The approach is intended to create a bridge between an academic perspective on what is legitimate and how society is responding to behaviour in practice. This is particularly important in a new field like cyber security where cultural and legal norms have not yet been established. In such areas there are likely to be inconsistencies in the reasoning behind legal and governmental responses to scandals.

From a delivery perspective, the module seemed to be very effective overall. The material was enjoyable to present and the students responded well to it. Full attendance was common, interaction was good, and marks for both coursework and the examination were generally high, though not all students were successful.

Scores from the student evaluation questionnaires are above the target level set by the University. Table 1 gives the detailed figures for two questions relevant to engagement across the three years of delivery. Each entry is the average score for that question, with the two figures in brackets indicating the number of students who responded and the standard deviation for the score. The figures tend to suggest a significant improvement after the first year of delivery but the size of the class is small that year and low outlier scores from one student may have resulted from a misunderstanding of the 1–5 response scale.

**Table 1:** Summary module evaluation results

Evaluation question	2014–2015	2015–2016	2016–2017
The lecturer explained new terms, concepts and principles clearly	4.2 (10, 1.3)	4.8 (16, 0.4)	4.7 (21, 0.5)
The lecturer made the subject interesting	4.2 (10, 1.4)	4.7 (16, 0.6)	4.8 (21, 0.6)

Some of the comments accompanying the scores supported the initial premise in this paper that students tend to have negative preconceptions about professional ethics. Comments include “Potentially very dry subject material made interesting” and “Very engaging and interesting module. Was much better than I expected it would be.” Students were very positive about the use of relevant real-world case studies and the pragmatic approach to understanding and handling ethical issues. Responses such as “Actually enjoyed the work and made the students more engaged” and “The lecturer is teaching the module in an enjoyable way” also suggest that the attempt to be entertaining, as a way of improving engagement, was beneficial.

The only negative comments received were in 2014–15, in relation to aspects of the coursework. Adjustments have been made since then, including a reversal of the coursework weighting with the examination, making coursework now worth 60%, up from 40% in preceding years.

Despite this encouraging feedback, an analysis of coursework results highlights some areas where improvement seems desirable. Considering the three pieces of coursework individually:

### Scandal Investigation

Each year about a third of the class produce excellent work for this exercise, with a small number suggesting unexpected motives and dynamics. The middle third find the task more challenging and tend to focus on “the facts of the case,” with much less emphasis on motives and dynamics. The final third tend to be students who are uncomfortable challenging an official narrative of events. These students typically identify an authority, such as the government or police, and recount their statements of events unchallenged without any discussion of other organisations that may have had an influence on the situation.

### Privacy Impact Assessment

Most students do reasonably well in this exercise, but there are two common sources of difficulties. The first is that some ignore the questionnaire results from their team members, thereby missing some of the most important risks associated with their products. The second difficulty is in students concentrating on familiar cyber security technical issues giving much less attention to privacy problems. A small number will produce solutions that focus almost entirely on avoiding external hacking. Possibly the most difficult students to assess are those whose work is competent but they show insufficient concern for the feelings of others. Typically such students will make proposals in their privacy impact assessments that are the most oppressive possible while remaining technically legal.

### Whistleblowing Scenario

Typically, the students took a range of different approaches to the construction of the whistleblowing email. Some took a submissive obedient line, some adopted a friendly informal style, some were confrontational, using a legal challenge approach, and some were conspiratorial, focusing on covering up the misconduct. The best students quoted real examples to show evidence that their strategies were plausible. Average students provide less evidence and focus more on their opinions, with the lowest group simply identifying legal risks or responsibilities and showing little or no appreciation for how others will judge their actions.

Improvement is possible by giving students more detailed guidance on what is being assessed and highlighting problematic responses. This may produce better results but the educational goal is to improve the thinking of the students involved. The work of Bairaktarova and Woodcock (2016) may be a way to address this issue. Their approach uses a motivational model for ethics that goes beyond teaching ethical judgement to include the assessment of students’ attitudes to ethical situations and their awareness of social norms and perceived controls on behaviour. This seems particularly relevant for the diverse group of cyber security students involved, who are from a range of different cultural backgrounds and have widely differing levels of work experience.

While improvements are possible, overall, the engagement oriented approach to teaching professional ethics seems to have been very effective in the particular circumstances described. The approach also seems sufficiently general to be applied to other technical subjects where professional ethics is presented as a full module.
